# Genetic Variability and Evolutionary Implications of RNA Silencing Suppressor Genes in RNA1 of *Sweet Potato Chlorotic Stunt Virus* Isolates Infecting Sweetpotato and Related Wild Species

**DOI:** 10.1371/journal.pone.0081479

**Published:** 2013-11-22

**Authors:** Arthur K. Tugume, Robert Amayo, Isabel Weinheimer, Settumba B. Mukasa, Patrick R. Rubaihayo, Jari P. T. Valkonen

**Affiliations:** 1 Department of Agricultural Sciences, University of Helsinki, Helsinki, Finland; 2 Department of Biological Sciences, School of Biosciences, College of Natural Sciences, Makerere University, Kampala, Uganda; 3 NARO-NaSARRI, Serere, Soroti, Uganda; 4 Department of Agriculture, College of Agricultural and Environmental Sciences, Makerere University, Kampala, Uganda; University of California, Riverside, United States of America

## Abstract

**Background:**

The bipartite single-stranded RNA genome of Sweet potato chlorotic stunt virus (SPCSV, genus *Crinivirus*; *Closteroviridae*) encodes a Class 1 RNase III (RNase3), a putative hydrophobic protein (p7) and a 22-kDa protein (p22) from genes located in RNA1. RNase3 and p22 suppress RNA silencing, the basal antiviral defence mechanism in plants. RNase3 is sufficient to render sweetpotato (*Ipomoea batatas*) virus-susceptible and predisposes it to development of severe diseases following infection with unrelated virus. The incidence, strains and gene content of SPCSV infecting wild plant species have not been studied.

**Methodology/Principal Findings:**

Thirty SPCSV isolates were characterized from 10 wild *Ipomoea* species, *Hewittia sublobata* or *Lepistemon owariensis* (family *Convolvulaceae*) in Uganda and compared with 34 local SPCSV isolates infecting sweetpotatoes. All isolates belonged to the East African (EA) strain of SPCSV and contained *RNase3* and *p7*, but *p22* was not detected in six isolates. The three genes showed only limited genetic variability and the proteins were under purifying selection. SPCSV isolates lacking p22 synergized with Sweet potato feathery mottle virus (SPFMV, genus potyvirus; *Potyviridae*) and caused severe symptoms in co-infected sweetpotato plants. One SPCSV isolate enhanced accumulation of SPFMV, but no severe symptoms developed. A new whitefly-transmitted virus (KML33b) encoding an RNase3 homolog (<56% identity to SPCSV RNase3) able to suppresses sense-mediated RNA silencing was detected in *I. sinensis*.

**Conclusions/Significance:**

SPCSV isolates infecting wild species and sweetpotato in Uganda were genetically undifferentiated, suggesting inter-species transmission of SPCSV. Most isolates in Uganda contained *p22*, unlike SPCSV isolates characterized from other countries and continents. Enhanced accumulation of SPFMV and increased disease severity were found to be uncoupled phenotypic outcomes of RNase3-mediated viral synergism in sweetpotato. A second virus encoding an RNase3-like RNA silencing suppressor was detected. Overall, results provided many novel and important insights into evolutionary biology of SPCSV.

## Introduction

Sweet potato chlorotic stunt virus (SPCSV; genus *Crinivirus*; family *Closteroviridae*) is a phloem-limited virus [1], semi-persistently transmitted by whiteflies [2,3] and known to occur in most areas where sweetpotatoes (*Ipomoea batatas* Lam.) are grown. The virus has a bipartite, single-stranded, positive-sense RNA genome [4]. Isolates can be identified as being the ‘East African’ (EA) and ‘West African’ (WA) strain by phylogenetic analysis of their *Hsp70h* gene located on RNA2 [5-7] and also by analysis of RNA1 sequences [8]. Strain EA is common in sweetpotatoes grown in East Africa and is also the only strain known to occur there [6,9]. There are only a few reports on its occurrence outside Uganda, e.g., in Kenya, Tanzania, China and Peru [7,8,10]. In contrast, strain WA occurs more widely in sweetpotatoes and has been detected in West and South Africa, Egypt, Israel, Spain, China, Peru, Argentina, and the United States [7,10-18]. 

The genome organization of SPCSV has many similarities to other criniviruses. Proteins implicated in virus multiplication are encoded by RNA1, whereas the genes for proteins needed for viral movement, encapsidation and vector transmission are located in RNA2 [19]. However, the genome of SPCSV possesses unique features especially concerning the gene content of RNA1. Downstream from the open reading frame (ORF) for the replicase, there is a 686 nucleotides (nt) long ORF for a Class 1 RNase III enzyme (RNase3, 27 kDa), an ORF (171 nt) for a putative, unique hydrophobic protein (p7), and an ORF (575 nt) for a 22-kDa protein (p22) [20]. These genes are not found in other *Closteroviridae* members and other RNA viruses. Furthermore, comparison of the fully characterized isolate SPCSV-Ug from Uganda, consisting of RNA1 (9407 nt) and RNA2 (8223 nt) [20], with isolate m2-47 from Peru shows that they are 98% identical, but m2-47 lacks the *p22* gene [8,10]. 

The 3’-proximal part of RNA1 constitutes an interesting genomic region for study owing to its unique gene functions. RNase3 and p22 are most likely expressed from the respective m^7^GpppN-capped subgenomic RNAs (sgRNA), which have been detected in SPCSV-infected plants [4]. RNase3 exhibits endoribonuclease activities similar to the Class 1 RNase III of *Escherichia coli* [20] and can suppress gene silencing induced by sense transcripts [21]. It is alone sufficient to eliminate antiviral defence to unrelated viruses in *RNase3*-transgenic sweetpotato plants and predisposes sweetpotato to development of the severe sweetpotato virus disease (SPVD) following infection with Sweet potato feathery mottle virus (SPFMV; Potyvirus, *Potyviridae*) [21]. On the other hand, the p22 protein is not needed for development of SPVD [8,21], although it is a ‘strong’ silencing suppressor. It can efficiently suppress gene silencing induced with dsRNA (hairpin RNA) and prevent cell-to-cell and long distance movement of the silencing signal when expressed at the site of silencing induction, something that RNase3 is unable to do [20]. It is plausible that p7 is expressed, but perhaps only temporarily and/or in low amounts from a non-capped sgRNA, as found with the 30K movement protein of *Tobacco mosaic virus* (genus *Tobamovirus*) [22]. The hydrophobic p6 protein of Beet yellows virus needed for intercellular virus transportation (genus *Closterovirus*) [23].

The 3’-proximal part of RNA1 has been characterized in 27 SPCSV isolates [4,8,10], all of which are from cultivated sweetpotato. Four isolates (from Argentina, Brazil and Israel) belong to the WA strain, whereas 23 isolates belong to the EA strain and include one isolate from Kenya, four isolates from Uganda, two isolates from Tanzania, and 16 isolates from Peru. Only the four EA strain isolates from Uganda contain *p22*. It seems reasonable to hypothesize that the variability in gene content (*p22*) is due to recombination-mediated gene loss or gain operating on RNA1. Furthermore, it would seem worthwhile to study whether or not *RNase3* and *p22* are subjected to evolutionary selective pressures adjusting their abilities to break down host resistance. Detection of molecular signatures of selection pressure on the putative p7 protein might provide information as to whether or not this gene (protein) is of importance to SPCSV. However, these issues could not be addressed comprehensively unless a representative collection of SPCSV was available from sweetpotato and the putative wild host species. Reports on natural infection of wild *Ipomoea* species with SPCSV are not available, but many wild *Ipomoea* spp. can be infected with SPCSV experimentally [24,25].

The aim of this study was to characterize isolates of SPCSV infecting wild species and sweetpotatoes in Uganda in order to understand evolutionary processes shaping the virus population. In East Africa, including Uganda, several biannual and perennial wild species of *Ipomoea* and other genera of *Convolvulacea* occur naturally, also as weeds in sweetpotato fields [26-28], but whether they are naturally infected by SPCSV has not been investigated. Therefore, a survey of SPCSV isolates in wild species and cultivated sweetpotatoes was carried out in Uganda and a number of the detected virus isolates were characterized for *RNase3*, *p7* and *p22* sequences. Whether the EA strain isolates of SPCSV show biological variability in terms of symptom induction and ability to synergise SPFMV in sweetpotato plants was also tested.

## Results

Wild plants of family Convolvulacea (genera *Ipomoea*, *Hewittia* and *Lepistemon*) were collected from a wide range of plant communities from different parts of Uganda in 2007, as previously described [28]. The wild plants sampled were mostly growing in close proximity to sweetpotato fields or grew as weeds in the field. The wild plants were identified using keys of Verdcourt [26], as described [28], and further characterized by DNA barcoding of the chloroplast gene *matK* [29]. Sweetpotato plants were also sampled from gardens in whose vicinity wild plants were collected. Furthermore, healthy seedlings of *I. setosa* were placed outdoors in shrubs next to a sweetpotato field in Mpigi district and were infected with SPCSV following natural transmission of the virus with whiteflies from the surrounding SPCSV infected wild plants or sweetpotatoes. Plants were established for further study by rooting cuttings in an insect-proof screenhouse at Makerere University Agricultural Research Institute, Kabanyolo (MUARIK).

### Phylogenetic Relationships of SPCSV Isolates

SPCSV was detected by TAS-ELISA from plants belonging to 12 wild species (*Ipomoea acuminata*, *I*. *cairica*, *I*. *obscura*, *I*. *repens*, *I*. *rubens*, *I*. *sinensis*, *I*. *spathulata*, *I*. *stenobasis*, *I*. *tenuirostris*, *I*. *wightii*, *Hewittia sublobata* and *Lepistemon owariensis*) growing in their natural habitats in 13 different geographical regions of Uganda. A total of 30 SPCSV isolates from different wild hosts and representing the 13 different regions (Table 1) were selected for further study along with 34 isolates of SPCSV detected in sweetpotatoes in the same 13 regions of Uganda. 

**Figure 1:**
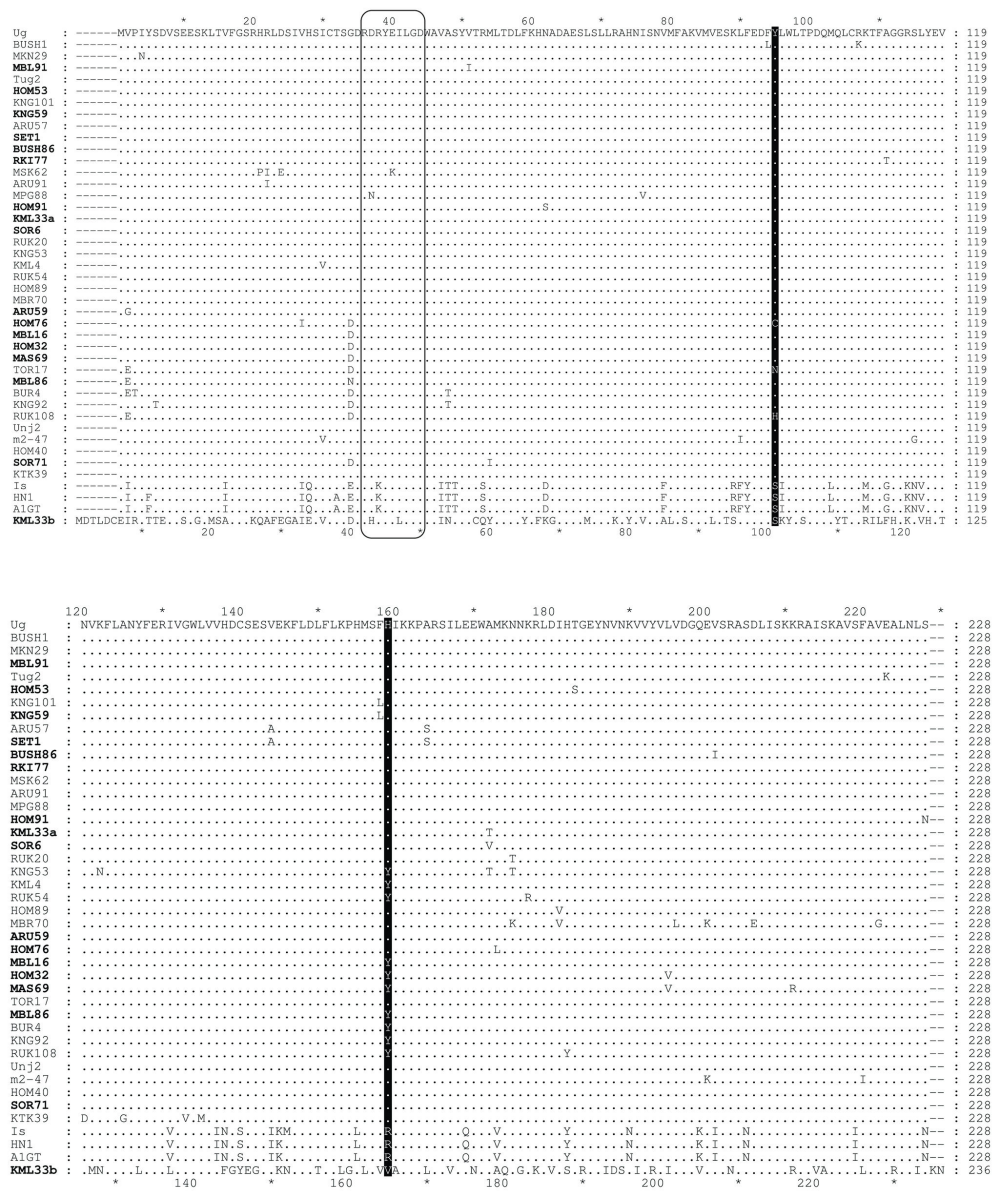
Alignment of the 43 different RNase3 protein amino acid (aa) sequences of *Sweet* *potato* *chlorotic* *stunt* *virus*. Groups of isolates containing identical RNase3 aa sequences are represented each by a single isolate. Numbers on top of the alignment indicate the aa positions with reference to isolate Ug (AJ428554) whereas numbers on the right indicate the number of the last amino acid at that position for each isolate. Numbers at the bottom of the alignment indicate aa positions in the new unknown virus (KML33b) related to SPCSV and detected in this study. The class 1 RNase III signature motif at aa positions 36-44 in SPCSV isolates (aa 42-50 in KML33b) is boxed. Two aa sites predicted to be under positive selection are indicated with black shades. Names of isolates characterized from wild plants are in bold.

Sequences of the 3’-proximal region of RNA1 in the SPCSV isolates were obtained by reverse transcription, amplification by PCR using a high-fidelity DNA polymerase and direct sequencing of the PCR products without cloning. The ORF for RNase3 was of identical length (684 nt; 228 aa) in all SPCSV isolates characterized in this study, including the 64 new isolates from Uganda, the two isolates detected in local sweetpotato cultivars in Central America (HN1 in Honduras and A1GT in Guatemala) [30] also characterized in this study, and the six previously characterized isolates from Tanzania, Uganda, Israel and Peru included for comparison (Table 1, Figure 1). The ORF for p7 was 171 nt (57 aa) in all EA strain isolates, but was shorter (165 nt; 55 aa) in the WA strain isolates. The ORF for p22 was of identical length (572 nt; 192 aa) in all isolates containing this gene. However, isolates KML33b, KTK40 (*I*. *sinensis*), SOR71 (*I*. *obscura*), HOM40, KTK39 and KTK41 (sweetpotato) characterized from Uganda and the two SPCSV isolates from Central America did not contain *p22*. Similarly, the previously characterized two EA strain isolates of SPCSV from Tanzania, one EA strain isolate from Kenya, the EA strain isolates from Peru and the WA strain isolates do not contain *p22* [8,10]. In all SPCSV isolates lacking *p22*, the gap in RNA1 was identically positioned. It began immediately after the stop codon for *p7* in the EA strain isolates and six nt downstream from the stop codon of *p7* in the WA strain isolates. 

**Table 1:** Sequence accessions of sweet potato chlorotic stunt virus (SPCSV) isolates characterized for their 3’ region of RNA1 from wild plants and cultivated sweetpotato in this study and those retrieved from the database previously characterized from cultivated sweetpotato.

Isolate**^*a*^**	Serotype**^*b*^**	Geographical origin	Sequence accession no.**^*c*^**	Host**^*d*^**	Habitat description**^*e*^**	Reference
ARU54*	EA	Arua, Uganda	GU127660	*I. sinensis*	Sweetpotato field	This study
ARU59	EA	Arua, Uganda	GU127659	*I. stenobasis*	Ornamental (20 m)	This study
BUSH2	EA	Bushenyi, Uganda	GU127638	*I. tenuirostris*	Sweetpotato field	This study
BUSH13	EA	Bushenyi, Uganda	GU127637	*I. cairica*	Grassland (200 m)	This study
BUSH42	EA	Bushenyi, Uganda	GU127639	*I. acuminata*	Shrubland (50 m)	This study
BUSH86	EA	Bushenyi, Uganda	GU127635	*I. tenuirostris*	Thicket (200 m)	This study
HOM32	EA	Hoima, Uganda	GU127650	*I. cairica*	Abandoned homestead (100 m)	This study
HOM53*	EA	Hoima, Uganda	GU127641	*H. sublobata*	Sweetpotato field	This study
HOM76*	EA	Hoima, Uganda	GU127653	*I. hederifolia*	Grassland (50m)	This study
HOM81	EA	Hoima, Uganda	GU127665	*I. acuminata*	Cassava-Maize intercrop (20 m)	This study
HOM91	EA	Hoima, Uganda	GU127633	*L. owariense*	Previous sweetpotato field (50 m)	This study
KAP88	EA	Kapchorwa, Uganda	GU127662	*I. obscura*	Grassland (10 m)	This study
KAP90	EA	Kapchorwa, Uganda	GU127613	*I. spathulata*	Shrubland (50 m)	This study
KML23*	EA	Kamuli, Uganda	GU127658	*I. sinensis*	Shrubland (10m)	This study
KML33a*	EA	Kamuli, Uganda	GU127657	*I. sinensis*	Sweetpotato field	This study
KML33b*	?	Kamuli, Uganda	GU127599	*I. sinensis*	Sweetpotato field	This study
KNG59	EA	Kanungu, Uganda	GU127629	*L. owariense*	Shrubland (50 m)	This study
KTK40*	EA	Katakwi, Uganda	GU127602	*I. sinensis*	Sweetpotato field	This study
MAS46*	EA	Masindi, Uganda	GU127661	*L. owariense*	Thicket (40 m)	This study
MAS52	EA	Masindi, Uganda	GU127655	*I. tenuirostris*	Disturbed habitat (5 m)	This study
MAS69*	EA	Masindi, Uganda	GU127652	*H. sublobata*	Grassland (70 m)	This study
MBL16	EA	Mbale, Uganda	GU127649	*I. acuminata*	Ornamental (100 m)	This study
MBL86	EA	Mbale, Uganda	GU127646	*I. acuminata*	Grassland (10 m)	This study
MBL91	EA	Mbale, Uganda	GU127666	*I. acuminata*	Disturbed habitat (5 m)	This study
RKI77	EA	Rakai, Uganda	GU127631	*I. cairica*	Sweetpotato field	This study
RUK106	EA	Rukungiri, Uganda	GU127640	*I. wightii*	Hedge (2 m)	This study
SET1*	EA	Mpigi, Uganda	GU127664	*I. setosa*	Shrubland (30 m)	This study
SET5*	EA	Mpigi, Uganda	GU127615	*I. setosa*	Shrubland (30 m)	This study
SOR6	EA	Soroti, Uganda	GU127616	*I. repens*	Grassland on a rock foot (500 m)	This study
SOR45*	EA	Soroti, Uganda	GU127618	*I. rubens*	Papyrus swamp (40 m)	This study
SOR71*	EA	Soroti, Uganda	GU127605	*I. obscura*	Abandoned sweetpotato field (80m)	This study
ARU57*	EA	Arua, Uganda	GU127663	sweetpotato	Sweetpotato field	This study
ARU91	EA	Arua, Uganda	GU127612	sweetpotato	Sweetpotato field	This study
BUR4	EA	Kanungu, Uganda	GU127647	sweetpotato	Sweetpotato field	This study
BUSH1	EA	Bushenyi, Uganda	GU127617	sweetpotato	Sweetpotato field	This study
HOM12	EA	Hoima, Uganda	GU127651	sweetpotato	Sweetpotato field	This study
HOM40	EA	Hoima, Uganda	GU127606	sweetpotato	Sweetpotato field	This study
HOM89	EA	Hoima, Uganda	GU127625	sweetpotato	Sweetpotato field	This study
KML4	EA	Kamuli, Uganda	GU127643	sweetpotato	Sweetpotato field	This study
KML77	EA	Kamuli, Uganda	GU127656	sweetpotato	Sweetpotato field	This study
KNG2*	EA	Kanungu, Uganda	GU127610	sweetpotato	Sweetpotato field	This study
KNG53	EA	Kanungu, Uganda	GU127632	sweetpotato	Sweetpotato field	This study
KNG92	EA	Kanungu, Uganda	GU127607	sweetpotato	Sweetpotato field	This study
KNG101	EA	Kanungu, Uganda	GU127628	sweetpotato	Sweetpotato field	This study
KTK39*	EA	Katakwi, Uganda	GU127604	sweetpotato	Sweetpotato field	This study
KTK41*	EA	Katakwi, Uganda	GU127603	sweetpotato	Sweetpotato field	This study
MBR2	EA	Mbarara, Uganda	GU127611	sweetpotato	Sweetpotato field	This study
MBR70	EA	Mbarara, Uganda	GU127630	sweetpotato	Sweetpotato field	This study
MKN29	EA	Mukono, Uganda	GU127654	sweetpotato	Sweetpotato field	This study
MPG88*	EA	Mpigi, Uganda	GU127622	sweetpotato	Sweetpotato field	This study
MSK7*	EA	Masaka, Uganda	GU127624	sweetpotato	Sweetpotato field	This study
MSK62*	EA	Masaka, Uganda	GU127623	sweetpotato	Sweetpotato field	This study
MSK84	EA	Masaka, Uganda	GU127627	sweetpotato	Sweetpotato field	This study
RKI7	EA	Rakai, Uganda	GU127636	sweetpotato	Sweetpotato field	This study
RKI15	EA	Rakai, Uganda	GU127621	sweetpotato	Sweetpotato field	This study
RKI51	EA	Rakai, Uganda	GU127620	sweetpotato	Sweetpotato field	This study
RUK20*	EA	Rukungiri, Uganda	GU127626	sweetpotato	Sweetpotato field	This study
RUK54	EA	Rukungiri, Uganda	GU127644	sweetpotato	Sweetpotato field	This study
RUK74	EA	Rukungiri, Uganda	GU127642	sweetpotato	Sweetpotato field	This study
RUK108	EA	Rukungiri, Uganda	GU127645	sweetpotato	Sweetpotato field	This study
SOR10	EA	Soroti, Uganda	GU127614	sweetpotato	Sweetpotato field	This study
SOR68*	EA	Soroti, Uganda	GU127619	sweetpotato	Sweetpotato field	This study
TOR14*	EA	Tororo, Uganda	GU127648	sweetpotato	Sweetpotato field	This study
TOR16*	EA	Tororo, Uganda	GU127609	sweetpotato	Sweetpotato field	This study
TOR17*	EA	Tororo, Uganda	GU127608	sweetpotato	Sweetpotato field	This study
Mis1	EA	Misungui, Tanzania	EU124493	sweetpotato	Sweetpotato field	Cuéllar et al. 2008
m2-47	EA	Peru	EU124490	sweetpotato	Sweetpotato field	Cuéllar et al. 2008
Tug2	EA	Mpigi, Uganda	EU124494	sweetpotato	Sweetpotato field	Cuéllar et al. 2008
Ug	EA	Mpigi, Uganda	AJ428554	sweetpotato	Sweetpotato field	Kreuze et al. 2002
Unj2	EA	Unguja, Tanzania	EU124492	sweetpotato	Sweetpotato field	Cuéllar et al. 2008
A1GT	WA	Guatemala	GU127601	sweetpotato	Sweetpotato field	This study
HN1	WA	Honduras	GU127600	sweetpotato	Sweetpotato field	This study
Is	WA	Israel	EU124491	sweetpotato	Sweetpotato field	Cuéllar et al. 2008

^a^ Isolates marked with asterisk (*) were experimentally transmitted by whiteflies to healthy sweetpotato plants of cv. Tanzania prior to molecular characterization.

^b^ EA, East African strain; WA, West African strain. Isolate KML33b does not belong to SPCSV and is marked with (?).

^c^ Publically available sequences of SPCSV characterized for their 3’ region of RNA1.

^d^ *I, Ipomoea*; *H, Hewittia*; *L, Lepistemon*.

^e^ The distance (in metres) between a sampled wild host plant and the nearest sweetpotato field is indicated.

Analysis of the RNA1 sequences using six different methods available in RDP v3.29 for detection of recombination breakpoints provided no evidence of recombination in any SPCSV isolate, as confirmed also with a PHI test (*p* = 0.999). 

**Figure 2:**
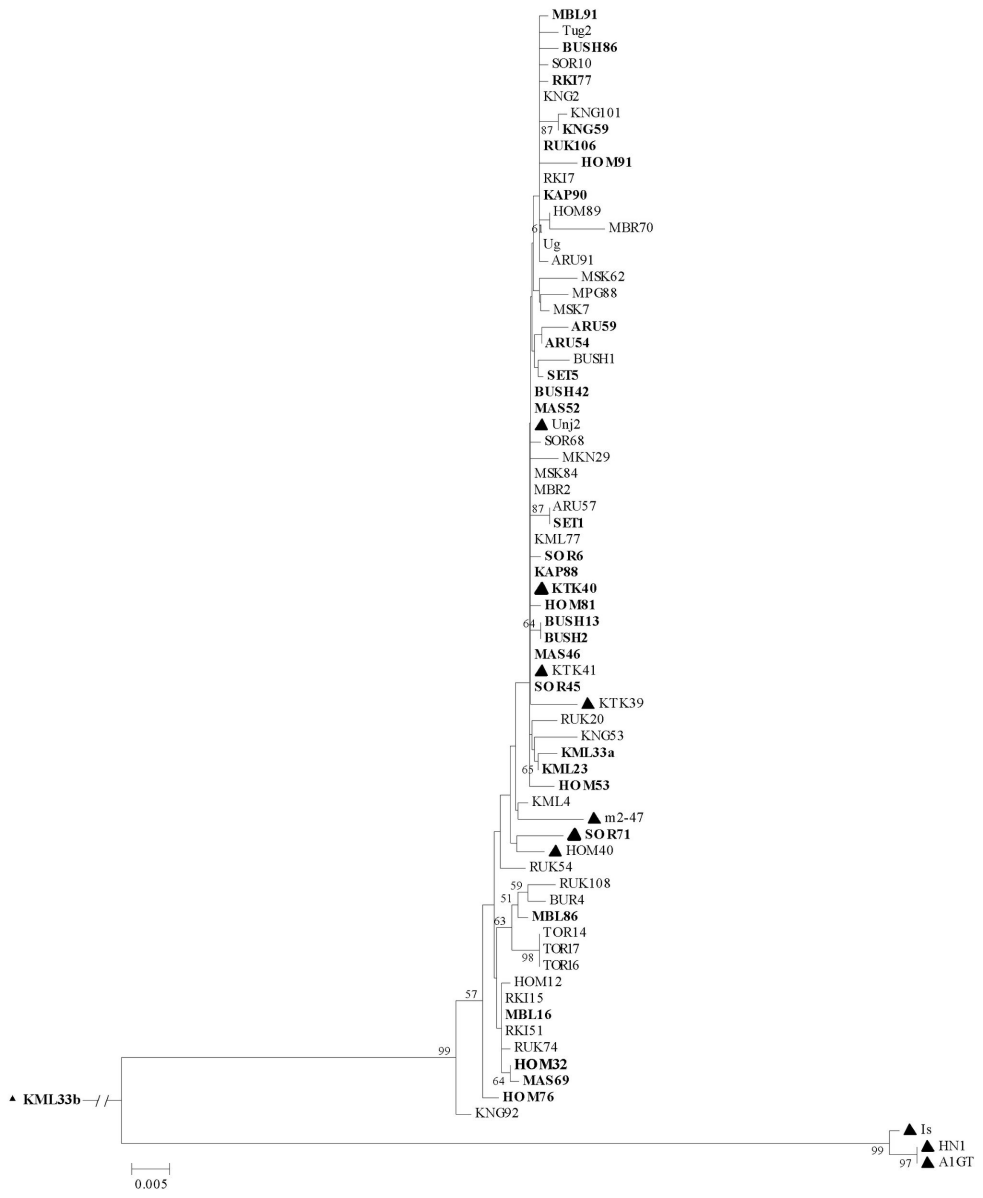
Phylogenetic analysis of genes coding for RNase3 of *Sweet* *potato* *chlorotic* *stunt* *virus* and the corresponding sequence of an unknown related virus (KML33b) detected in this study. The branch of KML33b is not fully depicted. Names of isolates characterized from wild plants are indicated in bold, whereas the ten SPCSV isolates lacking the p22 gene are indicated with a black triangle (▲). Numbers at branches represent bootstrap values of 1000 replicates. Only bootstrap values of ≥ 50% are shown. Scale indicates Kimura units in nucleotide substitutions per site [55].

The nt sequences of SPCSV isolates characterized in this study were subjected to phylogenetic analysis, including the previously characterized WA strain isolate from Israel and five EA strain isolates from Uganda, Tanzania and Peru for comparison. Results on *RNase3* sequences showed that all SPCSV isolates from Uganda belonged to the EA strain (Figure 2), no matter whether infecting wild species or sweetpotato. Only the two SPCSV isolates (HN1 and A1GT) from Central America belonged to the WA strain and formed a separate main clade with the WA isolate Is from Israel (Figure 2). Phylogenetic analysis of the *p7* gene sequences resulted in a similar grouping of isolates to the two SPCSV strains (Figure S1). Only few significant subclusters of two or three isolates were supported by high bootstrap values, and the subclusters differed depending on whether *RNase3* or *p7* was analyzed. Phylogenetic analysis based on the *p22* gene sequences revealed no significant subclusters (data not shown).

Irrespective of the gene used for analysis, there was no phylogenetic congruence of isolates with their host species, geographical origins in Uganda, and whether or not they encoded the *p22* gene.

### Nucleotide Diversity and Selection Pressure

Nt sequence identities of EA strain isolates ranged from 92.5 % (*p7*), 96.0% (*p22*) and 97.7% (*RNase3*) to 100 % depending on the gene analyzed. The aa sequence identities ranged from 93.1% (p7), 94.8% (p22) and 98.7% (Rnase3) to 100%. The p22 protein of isolate MBL16 (from *I*. *acuminata*) was distinguished from other isolates by 12 unique aa substitutions (Supporting information, Figure S2). The sequences of WA strain isolates from Honduras and Guatemala were identical to each other and nearly identical to the isolate Is; the nt (aa) sequence identities were 99.4% (98.7%) for RNase3 and 99.4% (100%) for p7. The EA and WA strain isolates showed nt (aa) sequence identities of 83.8-84.3% (81.6-82.5%) for RNase3 and 76.2-79.8% (60.7-66.7%) for p7.

Irrespective of the host species from which the SPCSV isolates were characterized, nt diversity (π) values for each of the three protein coding regions studied were relatively low being 2.2%, 4.3% and 0.71% for the *RNase3*, *p7* and *p22* gene, respectively. The non-synonymous nt diversity (π_a_) was 1.2%, 4.1% and 0.4% for the *RNase3*, *p7* and *p22* gene, respectively, whereas the synonymous nt diversity (π_s_) was 5.6%, 5.5%, and 1.6%, respectively. The value of π_a_ was 4.7 or 4.0 fold lower than the value of π_s_ for the *RNase3* and *p22* gene, respectively. The value of π_a_ (4.1%) was slightly lower than π_s_ (5.5%) for the p7 protein. The selection pressures suggested by the aforementioned results were assessed by a maximum likelihood framework of codon substitution under model M0 which yielded ω values of 0.268, 0.562 and 0.324 for the *RNase3*, *p7* and *p22* genes, respectively, indicating purifying selection (Table 2). Heterogeneity of selective pressure was revealed by a test using an M3 vs. M0 LRT which showed that M3 fitted the data significantly better than M0 (Table 2) for all proteins. M3 for *RNase3* suggested that 55.5% of sites were evolving under strong purifying selection (ω = 0.072), 43.9% of sites under weak purifying selection (ω = 0.513) and only 0.6% of sites under positive selection (ω = 7.781) (Table 2). The proportions of different site classes under M3 for the p7 and p22 genes are shown in Table 2. 

LRTs of nested models M2a vs. M1a showed that M2a was a better fit than M1a for RNase3, p7 and p22 proteins (Table 2) which is consistent with positive selection on some aa sites. Parameter estimates under M2a for the RNase3 showed a large proportion of sites (86.4%) under purifying selection (ω = 0.170), 13.1% under neutral evolution (ω = 1.000) and only 0.5% under positive selection (ω = 9.690). These results were verified in an independent LRT involving M8 vs. M7 in which M8 fitted the data for each gene better than M7 (Table 2). The Näive Empirical Bayes (NEB) inference provided similar results as BEB inference under M3, M2a and M8 (Table 2).

**Table 2:** Parameter estimates, log-likelihood (*ln*L) values, ω-ratio (*d*_N_/*d*_S_), and likelihood ratio test (LRT) statistics under six different maximum likelihood models of codon substitution that were used to investigate selection pressures exerted on the RNase3, p7 and p22 proteins encoded by the 3’ region of *Sweet potato chlorotic stunt virus* genomic RNA1.

			ω-ratio	Log-likelihood	LRT statistic**^*c*^**	Positively selected
Protein	Models**^*a*^**	Parameter estimates**^*b*^**	(*d*_N_/*d*_S_)	(*ln*L)	(2×δ*ln*L)	(amino acids) sites**^*d*^**
RNase3	M0	ω = 0.268	0,268	−2164.398		none
	M3	*p*_0_ = 0.555, *p*_1_ = 0.439 (*p*_2_ = 0.006)	0,309	−2149.580	*p* < 0.001	159H*
		ω_0_ = 0.072 ω_1_ = 0.513, ω_2_ = 7.781				
	M1a	*p*_0_ = 0.857 (*p*_1_ = 0.143), ω_0_ = 0.157 (ω_1_ = 1.000)	0,278	−2153.607		not allowed
	M2a	*p*_0_ = 0.864, *p*_1_ = 0.131 (*p*_2_ = 0.005)	0,323	−2150.100	*p* < 0.05	95Y, 159H*
		ω_0_ = 0.170, ω_1_ = 1.000, ω_2_ = 9.690				
	M7	*p* = 0.469, *q* = 1.158	0,287	−2155.577		not allowed
	M8	*p*_0_ = 0.995 (*p*_1_ = 0.005),	0,310	−2149.542	*p* < 0.01	95Y, 159H*
		*p* = 0.681, *q* = 1.835, ω_s_ = 8.556				
p7	M0	ω = 0.562	0,562	−824.805		none
	M3	*p*_0_ = 0.133, *p*_1_ = 0.832 (*p*_2_ = 0.035)	0,652	−808.709	*p* < 0.0001	32G**, 47C*
		ω_0_ = 0.000, ω_1_ = 0.527, ω_2_ = 5.996				
	M1a	*p*_0_ = 0.851 (*p*_1_ = 0.149), ω_0_ = 0.271 (ω_1_ = 1.000)	0,379	−817.282		not allowed
	M2a	*p*_0_ = 0.863, *p*_1_ = 0.102 (*p*_2_ = 0.035)	0,661	−808.994	*p* < 0.001	32G**, 47C
		ω_0_ = 0.403, ω_1_ = 1.000, ω_2_ = 6.015				
	M7	*p* = 0.823, *q* = 0.946	0,465	−817.970		not allowed
	M8	*p*_0_ = 0.965 (*p*_1_ = 0.035),	0,664	−809.176	*p* < 0.001	32G**, 47C
		*p* = 1.834, *q* = 2.099, ω_s_ = 6.123				
p22	M0	ω = 0.324	0,324	−1413.955		None
	M3	*p*_0_ = 0.890, *p*_1_ = 0.087 (*p*_2_ = 0.022)	0,414	−1400.157	*p* < 0.001	5A*, 31L, 160L, 161V**
		ω_0_ = 0.249, ω_1_ = 0.249, ω_2_ = 7.551				
	M1a	*p*_0_ = 0.880 (*p*_1_ = 0.119), ω_0_ = 0.170 (ω_1_ = 1.000)	0,269	−1407.356		not allowed
	M2a	*p*_0_ = 0.977, *p*_1_ = 0.000 (*p*_2_ = 0.022)	0,413	−1400.217	*p* < 0.001	5A*, 31L, 160L, 161V**
		ω0 = 0.249, ω_1_ = 1.000, ω_2_ = 7.538				
	M7	*p* = 0.419, *q* = 0.908	0,316	−1409.453		not allowed
	M8	*p*_0_ = 0.977 (*p*_1_ = 0.022),	0,415	−1400.196	*p* < 0.001	5A*, 31L, 160L, 161V**
		*p* = 33.174, *q* = 99.000, ω_s_ = 7.597				

^a^ The models are according to Yang et al. [57] (M0, M3, M7, M8), Wong et al. [59] and Yang et al. [58] (M1a, M2a).

^b^ The numbers of parameters for the different models were 1 (M0), 2 (M1a), 4 (M2a), 5 (M3), 2 (M7), or 4 (M8).

^c^ LRT statistics of M3 vs. M0 are tests of heterogeneity of selection pressures among codon sites, while M2a vs. M1a and M8 vs. M7 are tests of positive selection, all of which assess LRT statistic (2δ*ln*L) against a chi-square distribution with the degrees of freedom (d.f) equal to the difference in the number of parameters between the nested models under comparison.

^d^ Positively selected amino acid sites at posterior probabilities *P* > 95.0 (*) or *P* > 99 (**) are shown. Identification of amino acids under positive selection is based on Näive empirical Bayes (Neb) (under M3) or Bayes empirical Bayes (BEB) inference (under M2a or M8).

### Interactions Between SPCSV and SPFMV from Wild Plants in Sweetpotato

**Figure 3:**
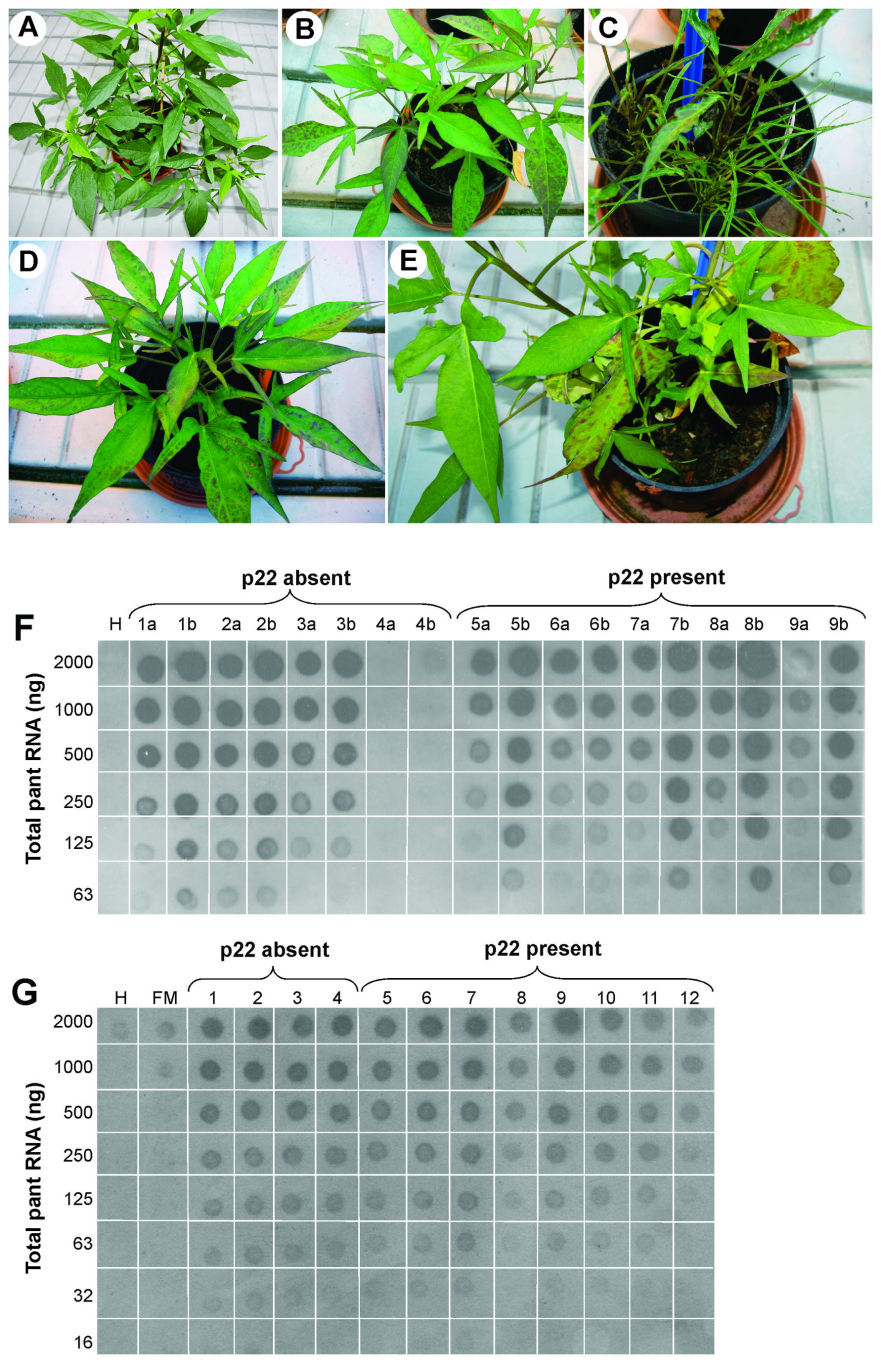
Symptoms and virus accumulation in single or double infections with Sweet potato chlorotic stunt virus (SPCSV) and Sweet potato feathery mottle virus (SPFMV) in sweetpotato plants of cv. Tanzania. (**A**) A plant infected with SPFMV (isolate RUK73) shows no obvious virus symptoms 6 weeks post-inoculation. (**B**) Chlorosis and purpling of older leaves induced by SPCSV isolate HOM76. (**C**) Typical symptoms of sweetpotato virus disease (SPVD) including retarded growth, severe leaf strapping and puckering induced by co-infection with SPCSV isolate HOM76 and SPFMV. (**D**) Chlorosis and purpling of older leaves induced by SPCSV isolate SOR71, and (**E**) similar symptoms in a plant co-infected with SOR71 and SPFMV. Young leaves develop normally in (D) SOR71-infected plants and (E) plants co-infected with SOR71 and SPFMV. In both cases the plants display only mild chlorosis typical of SPCSV infection, which indicates that SOR71 is not able to induce SPVD in co-infection with SPFMV. (**F**) SPCSV RNA detected by dot blot hybridization with a digoxigenin-labelled RNA probe specific to the *RNase3* gene in (a) plants infected with SPCSV alone or (b) plants co-infected with the SPCSV and SPFMV. The amounts of total plant RNA dotted on the membrane are indicated. The SPCSV isolates tested were 1, KTK39; 2, KTK40; 3, KTK41; 4, SOR71; 5, MAS69; 6, SET5; 7, MSK7; 8, TOR16; and 9, HOM76. Isolates 1 to 4 are lacking the p22 gene. Note that SOR71 (4a and 4b) accumulates at very low concentrations in sweetpotato leaves and is barely detectable. H, non-inoculated healthy plant of cv. Tanzania. (**G**) SPFMV RNA detected by dot blot hybridization with a digoxigenin-labelled RNA probe specific to the CP-encoding region. Samples 1 to 9 are those co-infected with SPCSV and SPFMV and tested for SPCSV (i.e., samples 1b to 9b) in (F). Three additional samples co-infected with SPCSV (10, MAS46; 11, TOR14; and 12, MPG88) and SPFMV were included. Note that SOR71 synergises SPFMV, which is detected by the enhanced SPFMV concentrations (sample 4) as compared to the samples (FM) from cv. Tanzania infected with SPFMV only. H, non-inoculated healthy plant of cv. Tanzania.

Twenty-four of those 64 isolates of SPCSV, which were characterized at the molecular level, had been initially transmitted with whiteflies from the original 12 wild plants and 12 sweetpotato plants sampled from the field to healthy plants of sweetpotato cv. Tanzania (Table 1). These wild plants belonged to seven species (*I. hederifolia*, *I. obscura*, *I. rubens*, *I. setosa*, *I. sinensis*, *Hewittia sublobata* and *Lepistemon owariensis*) which were amenable for establishment in the screenhouse using cuttings and were hence feasible for the experiment. The whitefly-inoculated plants of cv. Tanzania infected with SPCSV were multiplied by rooting stem cuttings and the new plants were observed for symptoms in the greenhouse. All isolates induced similar symptoms of mild chlorosis in younger leaves and purpling symptoms on older leaves, as shown for isolates HOM76 and SOR71 in Figures 3B and 3D, respectively. 

Subsequently, new cuttings were graft-inoculated with SPFMV isolate RUK73 (obtained from a plant of *I. obscura* in Uganda; [29]) in two experiments. All SPCSV isolates (except SOR71) induced the characteristic symptoms of SPVD including small, crinkled and strap-like leaves and severe retardation of plant growth in co-infected plants, as exemplified with coinfection of HOM76 and SPFMV in Figure 3C. Furthermore, three tested isolates lacking the *p22* gene (KTK39, KTK40 and KTK41) induced similar symptoms of SPVD as those 20 isolates which contained *p22*. 

Accumulation of SPCSV and SPFMV RNA was compared between the plants and treatments by dot blot hybridization using different amounts of total RNA extracted from the fully-grown upper leaves of cv. Tanzania. The viral RNA was detected with DIG-labelled RNA probes specific to *RNase3* and the CP-encoding region of SPFMV. The titres of SPCSV RNA1 in the leaves of plants co-infected with SPCSV and SPFMV were similar or slightly higher than those in plants infected with SPCSV alone (Figure 3F). The low concentrations of SPFMV RNA were barely detectable in plants of cv. Tanzania infected with SPFMV alone, but were greatly enhanced following co-infection with SPCSV, irrespective of the presence or absence of *p22* in the SPCSV isolate (Figure 3G). The relative differences in viral RNA concentrations were reproducible in the two experiments. 

**Figure 4:**
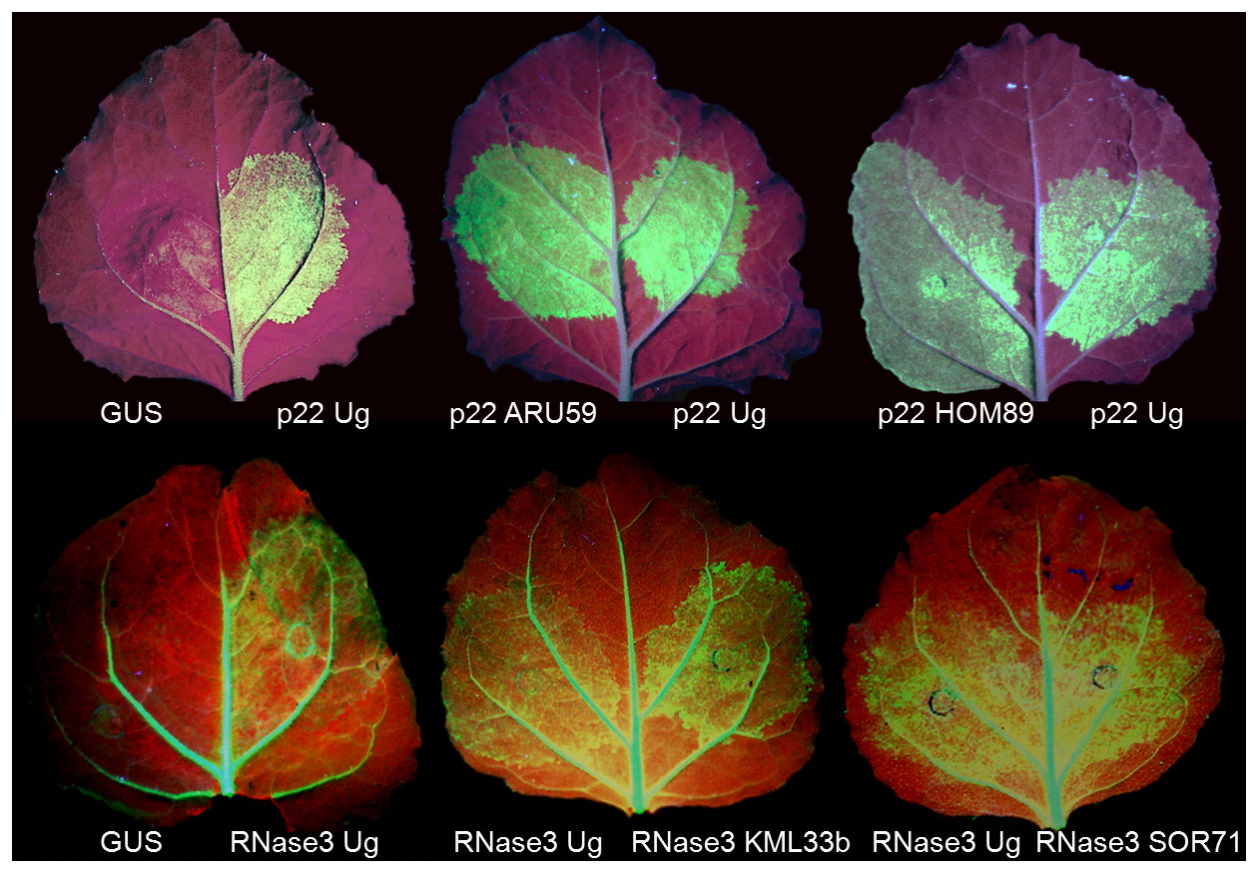
Suppression of RNA silencing by the p22 and RNase3 proteins of SPCSV isolates and the RNase3-like protein of the new virus KML33b. Upper row of leaves: “Silencing on the spot” to induce “strong silencing” of the *gfp* gene for green fuorescent protein (GFP) was achieved by co-expressing *gfp* from one *A. tumefaciens* strain and double-stranded (hairpin) RNA homologous to *gfp* from another strain in coinfiltrated leaf tissue of *Nicotiana benthamiana*, and coinfiltration of a third strain expressing p22 protein to suppress gfp silencing. The p22 proteins of isolates ARU59 (*I. sinensis*) and HOM89 (sweetpotato) were compared with the previously characterized p22 protein of isolate Ug by expressing them at the opposite sites of the midrib in the same leaf. An *Agrobacterium* strain expressing ß-glucuronidae (GUS) was included as the negative control. Leaves were illuminated with UV light and photographed from the underside with a digital camera 3 days postinfiltration. Lower row of leaves: Cosuppression of *gfp* in transgenic *N. benthamiana* plants (line 16c) constitutively expressing *gfp* (note the green fluorescence in veins). The spots were co-infiltrated with a mixture of two *Agrobacterium* strains, one expressing *gfp* to achieve cosuppression (silencing) of *gfp* and another expressing RNase3 of isolate SOR71 (*I. obscura*), Ug, or the RNase3-like protein of the new virus KML33b (*I. sinensis*). Leaves were illuminated with UV light and photographed from the underside with a digital camera 6 days postinfiltration.

Suppression of RNA silencing is supposed to increase virus titers in infected tissues, but virus concentrations were enhanced also in absence of p22 that is a strong suppressor of RNA silencing in SPCSV isolate Ug [20]. The p22 proteins of two new SPCSV isolates (ARU59 from *I. stenobasis* and HOM89 from sweetpotato) were tested for their ability to suppress gene silencing, including p22 of isolate Ug for comparison. Silencing of the jellyfish (*Aequorea victoria*) green fluorescent protein (GFP) gene expression was induced by double-stranded (hairpin) RNA in leaves of *Nicotiana benthamiana* using an *Agrobacterium tumefaciens* infiltration assay, as described [31]. All three p22 proteins supported GFP expression, i.e., suppressed *gfp* silencing, in contrast to expression of ß-glucuronidae (GUS) included as a negative control (Figure 4). These results were consistent with previous studies indicating that suppression of RNA silencing by p22 is dispensable for development of SPVD or enhancement of the titers of co-infecting viruses unrelated to SPCSV [21].

SPCSV isolate SOR71 was an exception, because the titers of this isolate both in single infection and double infection with SPFMV were too low to generate detectable signals within the time of exposure used (Figure 3F, samples 4a and 4b). Nevertheless, SOR71 was readily detected in these plants by RT-PCR and sequencing of the PCR products (*data not shown*). The plants co-infected with SOR71 and SPFMV (Figure 3E) did not develop SPVD symptoms, but symptoms remained mild and similar to those caused by SOR71 alone (Figure 3D). However, SPFMV titres were enhanced to the same extent as with other SPCSV isolates (Figure 3G, sample 4). 

Due to all these unexpected results on SOR71 (which lacks *p22*), RNase3 of SOR71 was tested for its ability to suppress RNA silencing using the aforementioned agroinfiltration assay. However, because RNase3 suppresses only sense-mediated gene silencing [21], i.e., caused cosuppression or “weak *gfp* silencing” [31], the *gfp* gene was overexpressed by agroinfiltration in leaves of transgenic *N. benthamiana* constitutively expressing *gfp* (line 16c) [32] and RNase3 of SOR71 was coexpressed in the same leaf tissue from another *A. tumefaciens* strain. RNase3 of the previously characterized SPCSV isolate Ug [20] and ß-glucuronidase (GUS) were included as a positive and a negative control, respectively. Results of the two experiments showed consistently that RNase3 of SOR71 and Ug supported expression of GFP fluorescence and suppressed sense-mediated silencing with similar efficiency (Figure 4).

### A New Putative Crinivirus encodes an RNase3-like Suppressor of Silencing

One plant of *I. sinensis* was co-infected with two distinct virus isolates, which were designed as KML33a and KML33b (Table 1). KML33a was nearly identical to other EA strain isolates of SPCSV (Figures 1 and 2). In contrast, the nt (aa) sequence of *RNase3* in KML33b was only 58.1-59.5% (53.0-55.6 %) identical to the EA strain isolates of SPCSV and 56.3% (50.9-51.3%) identical to the WA strain isolates (Figures 1 and 2). Furthermore, the RNase3 of KML33b was predicted to contain six additional aa residues at the N-terminus and two additional aa residues at the C-terminus, compared to that of SPCSV (Figure 1). The ORF for p7 was of identical length in KML33b and the SPCSV isolates, and the level of sequence similarity of p7 in KML33b and SPCSV isolates was comparable with the similarity of RNase3 nt and aa sequences. KML33b contained no ORF for p22. The corresponding gap in the genomic sequence of RNA1 started at an identical position but was 20 nt shorter as compared with SPCSV isolates. The low identity of KML33b to SPCSV and other viral sequences available in databases suggested that KML33b is a new virus related to viruses in genus *Crinivirus*.

The RNase3-like protein of KML33b was tested for its ability to suppress sense-mediated gene silencing (cosuppression), as described above. Leaves of the *gfp*-transgenic *N. benthamiana* line 16c were coinfiltrated with *Agrobacterium* strains for expression of *gfp* and KML33b RNase3 or GUS. Silencing was suppressed by KML33b RNase3, allowing continued GFP expression, whereas GFP fluorescence faded out by 6 days postinfiltration following infiltration with GUS (Figure 4). Hence, the KML33b RNase3 protein was able to suppress silencing. 

## Discussion

Twelve new natural host species for SPCSV were detected in this study. Natural occurrence of SPCSV in wild species has not been reported, but under experimental conditions, the WA strain of SPCSV is able to infect two of the species (*I. acuminata* and *I*. *wightii*; [24], which were found infected with EA strain isolates of SPCSV in the wild in Uganda.

Analysis of the *RNase3*, *p7* and *p22* gene sequences in a large number of SPCSV isolates from 12 wild species and sweetpotatoes in Uganda revealed nearly identical nt diversity indices and provide little phylogenetic evidence for diversification of the virus population. The observed similarities between virus isolates and lack of phylogenetic congruence with their wild and cultivated hosts [33,34] suggested that SPCSV isolates in Uganda are exchanged frequently between hosts in the field via transmission by the whitefly vectors. Similar observations have been made for Sweet potato mild mottle virus (SPMMV; genus *Ipomovirus*; Potyviridae) [35] and SPFMV [29] in the wild species of Convolvulaceae and cultivated sweetpotatoes in Uganda, Rice yellow mottle virus (RYMV; genus *Sobemovirus*) in cultivated rice and wild graminaceous species in East, Central and West Africa [36], and *African cassava mosaic virus* and *East African cassava mosaic cameroon virus* (genus *Begomovirus*; Geminiviridae) in cassava and various wild hosts in west Africa [37]. Furthermore, no isolate of the WA strain of SPCSV was detected in Uganda, which is consistent with previous studies on SPCSV in East Africa [5,6,9,10]. The two SPCSV isolates detected in sweetpotato samples from Central America belonged to the WA strain and are the only isolates besides the four isolates from Argentina, Brazil and Israel [8,10] which have been characterized for RNA1 sequences. 

Purifying selection was implicated on the majority of amino acids in SPCSV RNase3, which may be important for maintaining the reactive sites as well as the conformation necessary for enzymatic activities as proposed for some non-viral RNase III enzymes [38]. However, two amino acids were under positive selection in RNase3. They could be functionally important because the residue at position 159 is located in the C-terminal double-stranded RNA binding domain (dsRBD) and the other one at position 95 is in the N-proximal catalytic endoribonuclease domain (endND) of Class1 RNase III enzymes [39,40] (Figure 1). Hence, positive selection might translate into more efficient dsRNA binding and/or dsRNA cleavage, e.g., targeting different types of double-stranded small RNAs which RNase3 is able to cleave [21]. It is noteworthy that the positively selected amino acid residues 95Y and 159H in RNase3 found in the majority of EA strain isolates are not encoded by the three isolates of the WA strain but replaced by serine and arginine, respectively, in these isolates. The significance of this difference may become apparent with more isolates of the WA strain analyzed in future. 

The important function known for SPCSV RNase3 is its ability to eliminate antiviral defence, which also predisposes sweetpotato plants to development of severe disease symptoms following co-infection with heterologous viruses [21]. SPVD is the most harmful example of the viral synergism mediated by RNase3 and manifests in sweetpotato plants co-infected with SPCSV and SPFMV. This study demonstrated that isolates of SPCSV infecting wild plant species incite SPVD when transmitted to SPFMV-infected sweetpotato. However, isolate SOR71 detected in *I*. *obscura* was exceptional in that it did not incite SPVD symptoms although accumulation of SPFMV was enhanced to similar levels as with other SPCSV isolates in co-infected plants. The low titers of SOR71 were not the likely reason for its deviance from other isolates in induction of SPVD because quite minute titers of SPCSV are sufficient to enhance accumulation of SPFMV and induce SPVD, as shown in transgenic sweetpotatoes exhibiting highly efficient RNA-silencing based resistance against accumulation of SPCSV [41]. The results with SOR71 are very significant because they show that enhancement of SPFMV titer can be uncoupled from induction of SPVD. 

Because experimental evidence shows that RNase3 alone is responsible for the two phenotypic outcomes of the SPCSV-mediated synergism [21], the unique response in SOR71-infected sweetpotatoes could be linked to unique features of RNase3 in SOR71. However, the only difference which distinguishes the RNase3 of SOR71 from other SPCSV isolates is that a methionine residue is replaced with an isoleucine at position 54 in the catalytic domain adjacent to the RNase III signature motif (Figure 1). The data suggest a hypothesis on a dual mechanism by which the SPCSV-SPFMV synergism might be induced. The RNA-binding activity of RNase3 may contribite to the silencing suppression enforced by the HC-Pro protein of SPFMV, and the additive effects of the two silencing suppressors may overcome antiviral defence which limits accumulation of SPFMV in cv. Tanzania. This scenario is supported by siRNA binding being a common strategy for silencing suppression by viral proteins, including HC-Pro [42,43]. The catalytic activity of RNase3 shown to be required for silencing suppression might, in turn, manipulate or modulate cellular RNA biogenesis and homeostasis to facilitate accumulation of SPCSV to the typical titers in sweetpotato, something that SOR71 fails to do. RNase3 activity in sweetpotato cells seems to be specifically targeted to a component of the antiviral defence system because plants over-expressing RNase3 display no discernible developmental or physiological phenotypes, except loss of resistance to viruses [21]. Therefore, the severe symptoms of SPVD which significantly differ from the much milder symptoms caused by SPCSV may be an ‘accidential’ consequence of the general loss of antiviral defence caused by RNase3, which allows other viruses disturb the physiology of co-infected plants in a manner which they alone are not able to. 

A novel virus isolate carrying predicted ORFs for proteins homologous to the RNase3 and p7 of SPCSV was detected in *I. sinensis* and designated as KML33b. It was found co-infecting the wild plant with an EA strain isolate of SPCSV and both were transmitted by whiteflies to healthy sweetpotato cv. Tanzania. The sequences of KML33b were highly divergent from SPCSV isolates and showed < 60% nt and aa sequence identity. The RNase3-like protein of KML33b was able to suppress sense RNA-mediated gene silencing, similar to RNase3 of SPCSV. The finding is significant because, so far, SPCSV was the only RNA virus known to carry a gene for an RNase III-like protein that suppresses RNA silencing [8,20,21]. Other genomic and biological features of KML33b remain a topic for future study. 

Positive selection detected on residues 32G and 47C of the p7 protein suggests that it is important at some stage of the viral infection cycle and may correspond to host adaptation of SPCSV. The functions of the p7 protein in SPCSV genome are not well understood, which hampers more specific attempts to predict the biological significance. The two aa positions occupy hydrophobic residues in all isolates characterized and hence contribute to hydrophobicity which is characteristic to many small proteins encoded by members of family *Closteroviridae* [19]. Presuming that p7 is involved in viral cell-to-cell movement, similar to the hydrophobic p6 protein of Beet yellows virus (*Closterovirus*) [23,44], positive selection of specific residues of p7 may occur to adapt the virus to, e.g., the multitude of different host species available for SPCSV in Uganda.

The *p22* gene was present in the majority and absent only from few (7.7%) EA strain isolates characterized from Uganda. This finding is intriguing, because previous studies comparing 23 EA strain isolates from Uganda, Tanzania, Kenya and Peru found *p22* only in the four Ugandan isolates [8,10]. Furthermore, the WA strain isolates from Israel and South and Central America did not contain *p22*. No molecular signatures for recombination were detected in the studied region of RNA1. Moreover, SPCSV isolates showed no phylogenetic congruence for the *RNase3* and *p7* genes according to whether or not they encode p22, indicating that variability in *RNase3* and *p7* and acquisition of *p22* have been driven by independent evolutionary processes. Positive selection was implicated on four aa sites of p22, which may indicate some measure of selective advantage in RNA silencing suppression or other yet unidentified functions of p22 in the EA strain. Purifying selection was found on 97.8% of the amino acids of p22 and has also been implicated on the evolution of other silencing suppressor proteins such as p23 and p20 encoded by *Citrus tristeza virus* (genus *Closterovirus*) [45]. Taken together, these data support the hypothesis that p22 provides an advantage to the EA strain isolates prevailing in Uganda. Data about the gene content in RNA1 of SPCSV in other countries of the Lake Victoria zone in East Africa is lacking almost completely and the incidence of SPCSV isolates containing *p22* remains a subject of an interesting future study. However, in light of the current information, it seems likely that SPCSV has acquired *p22* from an unknown organism in East Africa.

The current study has extended the knowledge of genetic variability and evolution of SPCSV by including many isolates from previously unknown wild host species in East Africa and characterizing a large number of SPCSV isolates for the 3’-terminal part of RNA1, which encodes proteins of conceivable importance for viral virulence. Results on selective pressures directed on the studied ORFs provided evidence that RNase3, p7 and p22 are mainly under purifying selection. Positive selection on a few amino acids in each protein implied that SPCSV isolates are under continuous adaptation, presumably due to the large diversity of hosts they infect in the field. The study of viral diversity in the wild species also revealed a previously unknown virus which is related to SPCSV and carries an RNA silencing suppressor homologous to RNase3. Furthermore, experiments using one SPCSV isolate from wild species demonstrated that the two phenotypic outcomes of synergism between SPCSV and SPFMV, namely enhancement of SPFMV accumulation and development of severe symptoms, can be uncoupled. These data provide novel understanding on evolution of the genetically diverse viruses in *Closteroviridae* and highlight the value of studying virus isolates from a wide range of natural hosts. 

## Materials and Methods

### Collection of Virus Isolates

Wild plants of family Convolvulacea were collected in 2007, identified and maintained as described by Tugume et al. [28,29,35]. Samples were collected from private land with the owner’s permission. For any other locations, no specific permissions were required for sampling. The field studies did not involve endangered or protected species.

Isolates of SPCSV from 13 wild plants and 12 plants of cultivated sweetpotato were whitefly-transmitted to healthy sweetpotato cv. Tanzania at MUARIK. Whiteflies (*Bemisia tabaci* Gennadius) were raised and maintained on cassava (*Manihot esculenta* Crantz) plants in insect-proof cages for three weeks. Single source plants infected with SPCSV were placed into insect-proof cages and 70-100 adult whiteflies were transferred to each. After a 36-h access period, a 3-week old healthy sweetpotato plant cv. Tanzania (raised from a cutting) was added into the cage for 72 h to be inoculated by the whiteflies and then treated with pymetrozine (Fulfill; Syngenta, Greensboro, NC) to kill the whiteflies. The inoculated sweetpotato plants were tested for SPCSV after 3 wk. Stem cuttings were taken and transferred to University of Helsinki, Finland, where they were grown in an insect-proof greenhouse (temperature 25-30 °C, relative humidity 70%) under natural daylight extended to 16h by illumination with high-pressure sodium halide lamps (light intensity of 150-200 μmol s^-1^m^-2^).

### Serological and Molecular Detection of SPCSV

Triple antibody sandwich enzyme-linked immunosorbent assay (TAS-ELISA) was used for serological detection of SPCSV as described [46]. Polyclonal and monoclonal antibodies to SPCSV were provided by the International Potato Centre (Lima, Peru) and Julius Kühn Institute (Braunschweig, Germany), respectively. Testing was repeated on the plants established in the screenhouse. The SPCSV in wild plants was graft-transmitted to healthy sweetpotato plants of cv. Tanzania for ease of maintenance and study. The presence of SPCSV in plants grown from the tuberous roots obtained from Central America was tested using molecular methods.

Total RNA was extracted using TRIzol Reagent (InVitrogen Ltd, UK) according to the manufacturer’s instructions from 200 mg of leaf tissue. First-strand cDNA was synthesized on 3 µg of total RNA with random primers and *Moloney murine leukemia virus* (M-MuLV) reverse transcriptase RNase H^-^ (Finnzymes Oy, Finland) according to the manufacturer’s instructions. The 3’ genomic region of the RNA1 of SPCSV was amplified by polymerase chain reaction (PCR) using a forward CSVR3-F2 (5’-GTGTTTCATACATTGTTTGTGTGCT-3’) and a reverse CSVp22-R2 (5’-AGGTGTATGACTCTAGGGTATAAAC-3’) primers designed in this study and which were complementary to the genomic positions 7491-7515 and 9241-9265, respectively, of the SPCSV EA strain RNA1 (AJ428554; Kreuze et al., 2002). The primers PolymF4b (5’-CGAATACGTGGTTGTCAA-3’) and WA-R1 (5’-CTCTAGGATACAAACATTAATC-3’) complementary to genomic positions 7046-7063 and 1270-1291 of SPCSV RNA1 accessions AJ428554 and EU124491, respectively, were used to amplify the 3’ genomic region of two SPCSV isolates from Central America. The PCR mixture and cycling parameters were those recommended for Phusion High-Fidelity DNA Polymerase (Finnzymes Oy, Finland).

### Sequence Analyses

The PCR products were purified using a combination of Exonuclease I and Calf Intestine Alkaline Phosphatase (Fermentas) as recommended [47]. The purified PCR products were sequenced directly with the Big Dye Terminator kit version 3.1 on an ABI automatic 3130 XL Genetic Analyzer at Haartman Institute, University of Helsinki, using the PCR primers. Sequencing was done in both directions for each of the two independent amplicons of each isolate. The sequences were compared by BLAST search with the existing sequences available in the NCBI database.

Nucleotide (nt) sequences were aligned using CLUSTALX version 1.83 [48], and examined visually. The ORFs for the *RNase3*, *p7* and *p22* genes of SPCSV were translated into amino acid (aa) sequences using the EMBOSS tool (http://www.ebi.ac.uk/emboss/transeq/index.html?). Percent nt and aa identities between sequences were computed using the CLUSTALW procedure [49] as implemented in MEGALIGN program of the DNASTAR software package (DNASTAR Inc., Madison WI, USA).

Average nucleotide diversity (π), the number of nonsynonymous nt substitutions per nonsynonymous site (*d*_*N*_) and the mean of dN values in all pairwise comparisons of the sequences of the gene (i.e., the nonsynonymous nucleotide diversity, symbolized π_*a*_), and the number of synonymous nt substitutions per synonymous sites (*d*_*S*_) and the mean of dS values in all pairwise comparisons of the sequences of the gene (i.e., the synonymous nucleotide diversity, symbolized π_*s*_) for each of the genes *RNase3*, *p7*, and *p22* were calculated using DnaSP version 5 [50]. The sequence data were analyzed for recombination breakpoints using RDP, Maximum Chi square, Bootscan, Chimaera, and Sister Scan (Recombination Detection Program RDP3 Package version 3.29; [51]) and verified using the Pairwise homoplasy index (PHI) test for recombination [52]. 

Phylogenetic relationships based on the *RNase3*, *p7* and *p22* genes were analyzed using the Neighbor-joining algorithm [53] as implemented in MEGA4 [54] using the Kimura two-parameter nucleotide substitution model [55]. Statistical significance of tree branching was tested by performing 1,000 bootstrap replications.

### Analysis of Selection Pressures

Non-synonymous to synonymous nt substitution rate ratio (ω) which gives a measure of selection pressure at the protein level was assessed using maximum likelihood codon substitution models implemented in CODEML programme of PAML4 package [56]. Six site models including M0 (one-ratio), M1a (nearly neutral), M2a (positive selection), M3 (discrete), M7 (beta), and M8 (beta&ω) were employed as described [57-59]. Likelihood ratio tests (LRTs) of M3 vs. M0 were used to test the heterogeneity of selective constraints among codons, while two comparisons of M2a vs. M1a, and M8 vs. M7 were used to test for positive selection as described [59]. Where the LRTs suggested positive selection, the Bayes empirical Bayes (BEB) approach [58] was used to identify amino acids under positive selection.

### Analysis of Interactions Between SPCSV and SPFMV in Cultivated Sweetpotato and Accumulation of Viruses

Scions of sweetpotato cv. Tanzania singly infected with SPFMV EA strain isolate RUK73 (FJ795761) isolated from *I. obscura* in Uganda [29] were grafted onto 20 sweetpotato plants of cv. Tanzania raised from cuttings taken from the rootstocks that had been inoculated with different isolates of SPCSV via whitefly transmission. The grafted plants were maintained as above and observed for SPVD symptoms. 

Concentrations of SPFMV and SPCSV (RNA1) were estimated by dot blot hybridization analysis, using digoxigenin (DIG) labeled RNA probes for the CP encoding region of SPFMV isolate RUK73 or the *RNase3* gene of SPCSV isolate HOM76 (Table 1), respectively. The RNA probes were synthesized by *in vitro* transcription of the cloned DNA fragments encoding these genes as recommended in the DIG Application Manual for Filter Hybridization (Roche, Mannheim, Germany). Total RNA was extracted from the sixth leaf below the uppermost fully expanded leaf at 6 wk after planting the SPCSV-infected cuttings or 6 wk after top graft-inoculation of SPFMV onto SPCSV-infected rootstock. The quantity and quality of the RNA were measured using a spectrophotometer and the concentrations of RNA samples were equalized. A dilution series was prepared from total plant RNA starting from 2000 ng/μL and 5 µl from each RNA dilution was dotted on positively charged nylon membrane (Hybond-N+, Amersham Bioscience Ltd, UK) and detected using the digoxigenin-labelled RNA probes following the procedures described in the DIG Application Manual for Filter Hybridization (Roche).

### Agroinfiltration Assays on Suppression of RNA Silencing

The experiments testing suppression of RNA silencing by p22 and RNase3 were carried out essentially as described by Kreuze et al. [20] and Cuéllar et al. [21]. *Agrobacterium tumefaciens* (strain C58c1; pGV3850) was transformed with binary vectors (pA35Shp200) each expressing one of the proteins under *Cauliflower mosaic virus* 35S promoter, and the 5’ untranslated region (5’UTR) of Potato virus A (PVA) was used for translation enhancement [20]. GUS was included as a negative control. Leaves of *N. benthamiana* were coinfiltrated with *Agrobacterium* strains for expression of the *gfp* gene for green fluorescent protein (GFP), *gfp*-specific double-stranded (hairpin) RNA to induce “strong gfp silencing” [31], and a third construct to express the putative silencing suppressor, as described previously [20]. Alternatively, leaves of transgenic *N. benthamiana* plants (line 16c) constitutively expressing *gfp* [32] were co-infiltrated with a mixture of two *Agrobacterium* strains, one expressing *gfp* to achieve cosuppression (silencing) of *gfp* and another expressing the putative silencing suppressor protein. Cultures of *A. tumefaciens* were diluted to OD_600_=0.25 or OD_600_=0.50 for the two types of experiments, respectively. Plants were grown from seeds in growth rooms under controlled conditions (16 h photoperiod at 18/22 °C, 70% relative humidity; 200 μE m^–2^s^–1^ light intensity) and given fertiliser (0.3 g/l) (16:9:22 N:P:K; Yara, Espoo, Finland) at every watering. The first 3-4 fully expanded leaves were agroinfiltrated.

## Supporting Information

Figure S1 **Phylogenetic analysis of genes coding for p7 of *Sweetpotatochloroticstuntvirus* and the corresponding sequence of an unknown related virus (KML33b) detected in this study.** Names of isolates characterized from wild plants are indicated in bold, whereas the ten SPCSV isolates lacking the p22 gene are indicated with a black triangle (▲). Numbers at branches represent bootstrap values of 1000 replicates. Only bootstrap values of ≥ 50% are shown. Scale indicates Kimura units in nucleotide substitutions per site [55].
(PDF)

Figure S2 **Alignment of the 24 different p22 protein amino acid sequences of Sweet potato chlorotic stunt virus.** Groups of isolates containing identical p22 aa sequences are represented each by a single isolate. The aa sites predicted to be under positive selection (black shades) and the 12 unique aa substitutions in isolate MBL16 (arrows) are pointed out. Numbers on top of the alignment indicate the aa positions with reference to SPCSV isolate Ug (AJ428554). Names of isolates from wild plants are in bold.
(PPT)
