# Supplementary material for: Genetic Variability and Evolutionary Implications of RNA Silencing Suppressor Genes in RNA1 of Sweet Potato Chlorotic Stunt Virus Isolates Infecting Sweetpotato and Related Wild Species
Source: PLoS One. 2013 Nov 22;8(11):e81479. doi: 10.1371/journal.pone.0081479 (PMC3838340; doi:10.1371/journal.pone.0081479)

**Supporting information Figure S1.** Phylogenetic analysis of genes coding for (A) RNase3 and (B) p7 of Sweet potato chlorotic stunt virus and the corresponding sequences of an unknown related virus (KML33b) detected in this study. The branch of KML33b is not fully depicted in (A). Names of isolates characterized from wild plants are indicated in bold, whereas the ten SPCSV isolates lacking the p22 gene are indicated with a black triangle (▲). Numbers at branches represent bootstrap values of 1000 replicates. Only bootstrap values of ≥ 50% are shown. Scale indicates Kimura units in nucleotide substitutions per site (Kimura, 1980).

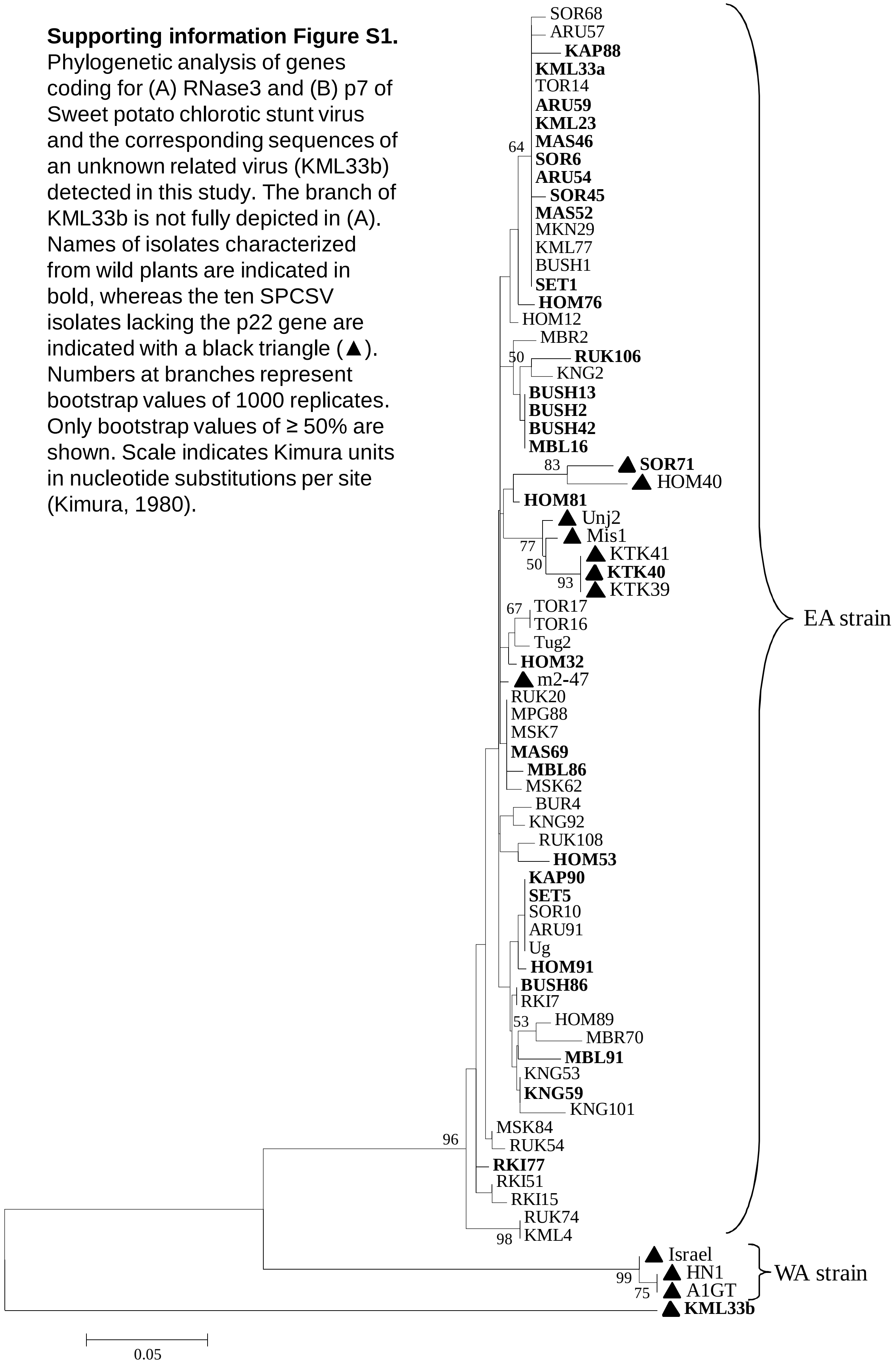

Supplement: Figure S1 — Phylogenetic analysis of genes coding for p7 of Sweetpotatochloroticstuntvirus and the corresponding sequence of an unknown related virus (KML33b) detected in this study. Names of isolates characterized from wild plants are indicated in bold, whereas the ten SPCSV isolates lacking the p22 gene are indicated with a black triangle (▲). Numbers at branches represent bootstrap values of 1000 replicates. Only bootstrap values of ≥ 50% are shown. Scale indicates Kimura units in nucleotide substitutions per site [55]. (PDF) [file pone.0081479.s001.pdf]
