# Supplementary material for: Genetic Variability and Evolutionary Implications of RNA Silencing Suppressor Genes in RNA1 of Sweet Potato Chlorotic Stunt Virus Isolates Infecting Sweetpotato and Related Wild Species
Source: PLoS One. 2013 Nov 22;8(11):e81479. doi: 10.1371/journal.pone.0081479 (PMC3838340; doi:10.1371/journal.pone.0081479)
Supplement: Figure S2 — Alignment of the 24 different p22 protein amino acid sequences of Sweet potato chlorotic stunt virus. Groups of isolates containing identical p22 aa sequences are represented each by a single isolate. The aa sites predicted to be under positive selection (black shades) and the 12 unique aa substitutions in isolate MBL16 (arrows) are pointed out. Numbers on top of the alignment indicate the aa positions with reference to SPCSV isolate Ug (AJ428554). Names of isolates from wild plants are in bold. (PPT) [file pone.0081479.s002.ppt]

## Slide 1
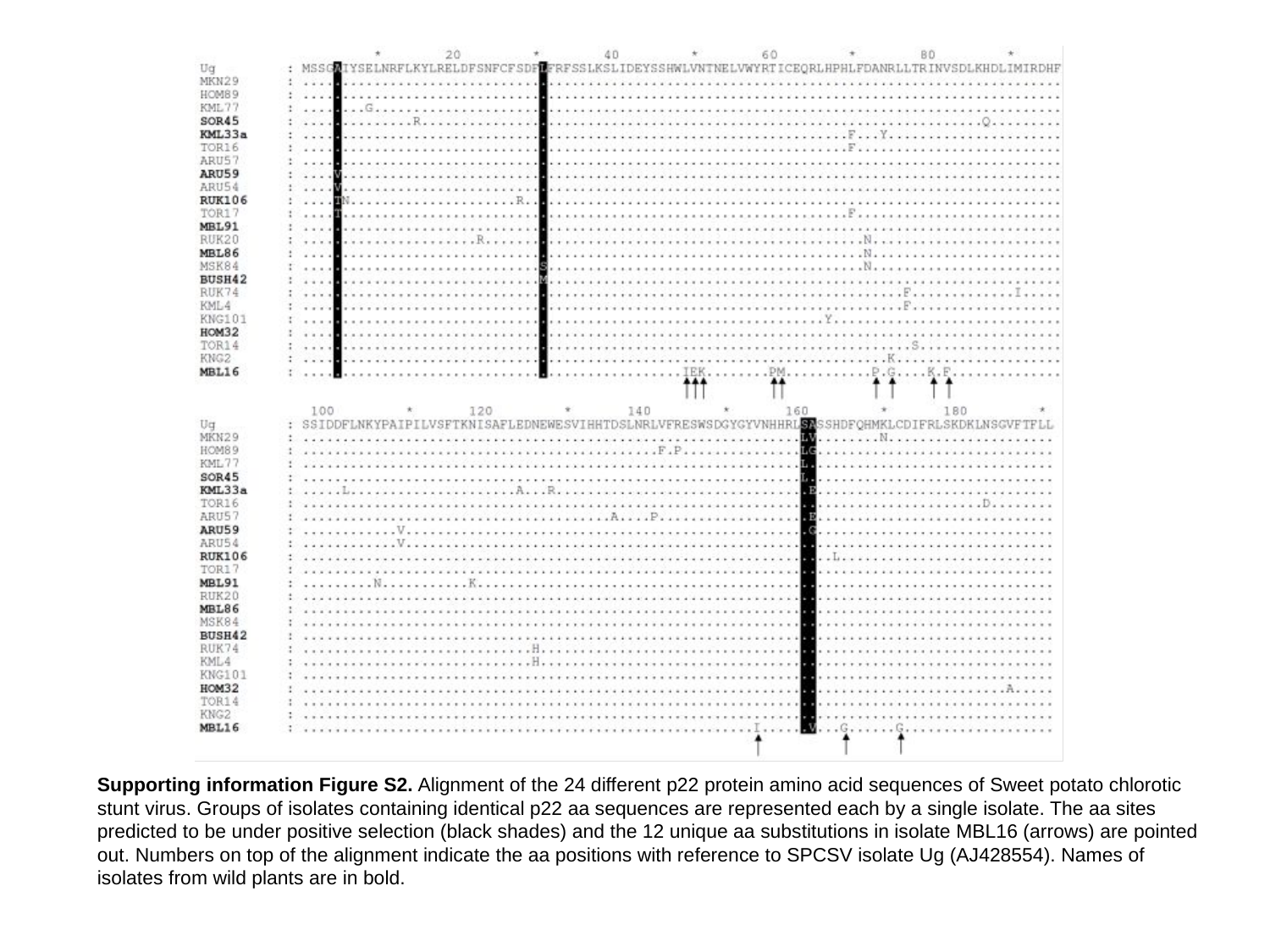

Supporting information Figure S2. Alignment of the 24 different p22 protein amino acid sequences of Sweet potato chlorotic stunt virus. Groups of isolates containing identical p22 aa sequences are represented each by a single isolate. The aa sites predicted to be under positive selection (black shades) and the 12 unique aa substitutions in isolate MBL16 (arrows) are pointed out. Numbers on top of the alignment indicate the aa positions with reference to SPCSV isolate Ug (AJ428554). Names of isolates from wild plants are in bold.
